# Machine Learning in Assessing Intraoperative Blood Loss: A Systematic Review and Meta‐Analysis

**DOI:** 10.1111/inr.70159

**Published:** 2026-02-27

**Authors:** Wenlin Zhou, Linglin Pan, Xinmei Pan, Yanwen Li, Lin Rao, Hong Li

**Affiliations:** ^1^ Department of Nursing The International Peace Maternity and Child Health Hospital, School of Medicine, Shanghai Jiao Tong University Shanghai China; ^2^ School of Nursing Shanghai Jiao Tong University Shanghai China; ^3^ Delivery Unit The International Peace Maternity and Child Health Hospital School of Medicine Shanghai Jiao Tong University Shanghai China; ^4^ Operating Theater The International Peace Maternity and Child Health Hospital School of Medicine Shanghai Jiao Tong University Shanghai China; ^5^ Shanghai Key Laboratory of Embryo Original Diseases The International Peace Maternity and Child Health Hospital, School of Medicine, Shanghai Jiao Tong University Shanghai China

**Keywords:** blood loss, Machine learning, meta‐analysis, surgical safety

## Abstract

**Aim:**

To evaluate the value of machine learning in assessing intraoperative blood loss by comparing associated outcomes with those of the gold standard.

**Background:**

Intraoperative bleeding is a leading cause of death in surgical patients and may be preventable through early and accurate assessment of blood loss. Machine learning models are used for measuring intraoperative hemorrhage with conventional assessment methods. However, outcome metrics vary across studies.

**Methods:**

A systematic review and meta‐analysis. Data were retrieved from Web of Science, PubMed, Embase, Cochrane Library, and CINAHL, with searches conducted through August 18, 2025.

**Results:**

Twelve studies were included. The pooled correlation coefficient between machine learning models and the gold standard for assessing intraoperative blood loss was high.

**Discussion:**

Machine learning models demonstrate high accuracy and reliability in assessing intraoperative blood loss. Heterogeneity was high, likely attributable to differences in publication year, country, study subjects, sample type, and modeling method.

**Conclusion:**

Models should be promoted for clinical use to improve blood loss assessment accuracy and to potentially reduce perioperative risk.

**Implications for Nursing:**

Novel machine learning models could enhance the accuracy and applicability of existing models, providing nursing staff with a more efficient tool for assessing blood loss. This will optimize the nursing decision‐making process, reduce adverse events caused by underestimating or overestimating blood loss, and improve patient safety.

**Implications for Nursing Policy:**

We provide a reference for exploring the application of artificial intelligence in other nursing fields, promoting interdisciplinary research and driving continuous innovation and progress in nursing.

## 1 Introduction

In 2017, the World Health Organization (WHO 2021) reported approximately 314 million surgical procedures worldwide and more than 4 million perioperative deaths. Many of these were attributable to massive intraoperative bleeding (Kietaibl et al. 2023; World Health Organization 2024). Massive hemorrhage is defined as blood loss exceeding total blood volume within 24 h, 50% of blood volume within 3 h, or >150 mL/min (Kietaibl et al. 2023). Inaccurate estimation of blood loss contributes to adverse outcomes, undermines clinical decision‐making, delays life‐saving interventions, and increases preventable morbidity and mortality worldwide (Shah et al. 2023). Several methods for estimating intraoperative blood loss exist; however, each has limitations. Visual estimation may misjudge blood loss by up to 65.4% (Townend and Byers 2018), especially in cases of large‐volume bleeding (American College of Obstetricians and Gynecologists Committee Opinion No. 794 2019). The weighing method, based on differences in absorbent material weight, is more accurate than visual estimation; however, contamination by non‐blood fluids often leads to overestimation (Gerdessen et al. 2021). The hemoglobin (Hb) method is frequently regarded as the “gold standard” (Atukunda et al. 2016) but involves logistical complexities that limit its real‐time applicability. Collectively, these shortcomings impede timely and appropriate interventions such as fluid resuscitation, transfusion, or return to surgery, thereby compromising patient safety (Liumbruno et al. 2023).

The WHO (2024) has identified “safe surgery” as a priority action area emphasizing that strengthening perioperative management and reducing surgical mortality are central goals in global efforts to improve healthcare quality and safety. Inaccurate blood loss estimation disrupts nursing workflows, impairs clinical judgment, and leads to misallocation of critical care resources (Shields et al. 2023). Overestimation of blood loss may result in unnecessary transfusions and increased costs, whereas underestimation increases the risk of inadequate tissue perfusion, shock, or death (Solon et al. 2013). Therefore, accurate and real‐time evaluation of intraoperative blood loss and the development of precise treatment strategies are essential for improving patient safety and reducing adverse events related to under‐ and overestimation of blood loss. Accurate assessment of blood loss volume is critical to improving the quality and safety of medical care and may be supported by machine learning (ML) approaches (Santosh et al. 2019). ML can integrate large and heterogeneous datasets to extract clinically relevant features, such as physiological signals, laboratory findings, and intraoperative imaging, to provide more robust, automated, and efficient blood loss predictions (Esteva et al. 2019; Chen et al. 2024). Preliminary studies using various algorithms, including support vector machines, random forests, and deep learning, have demonstrated improved accuracy of ML‐based approaches compared with conventional methods. Some ML models have been shown to estimate Hb levels in surgical sponges in real time with greater accuracy than the weighing method (Shi et al. 2024). Nowicki et al. used direct spectrophotometry as a reference method alongside ML, visual inspection, and weighing methods in a pediatric orthopedic surgery population and reported that ML‐based estimates were most strongly correlated with the reference standard (Nguyen et al. 2025). However, the accuracy of systems such as Triton's is limited by image quality (e.g., light and angle) and blood dilution (e.g., mixing with amniotic fluid), which may introduce measurement errors. Beyond measurement accuracy, ML represents a paradigm shift as a potential decision‐support tool that can be integrated into nursing workflows a (National Health Commission of the People’s Republic of China, 2024). By providing objective, real‐time, and automated estimates of bleeding volume, ML models may support timely clinical interventions, enhance decision‐making quality, optimize workflows, and reduce nursing workload, allowing greater focus on direct patient care and monitoring and thereby improving situational awareness in the operating room (Akbar et al. 2021; Kendale et al. 2023).

These models differ in algorithmic design, data type, feature selection, and validation metrics (Li et al. 2020, 2023; Doctorvaladan et al. 2017), leading to inconsistencies in the reported performance and clinical feasibility (Huang et al. 2021). Given this heterogeneity, a systematic evaluation of ML‐based intraoperative blood loss estimation is needed. This study used a meta‐analysis approach to systematically evaluate the relevant literature in this field, providing a reference for future model development and clinical applications.

## 2 Methods

### 2.1 Protocol and Registration

This study used previously published data and did not require additional ethical approval. This study was conducted according to the Preferred Reporting Items for Systematic Reviews and Meta‐Analysis (PRISMA) (Moher et al. 2010) and Meta‐analysis of Observational Studies in Epidemiology (MOOSE) (Stroup et al. 2000) guidelines. The protocol was registered with PROSPERO (CRD42025642792).

### 2.2 Question Constructing

The evidence‐based question was constructed using the PICO model:

Population: Surgical patients requiring assessment of blood loss (including orthopedic, general surgery, obstetrics and gynecology, and urology).
Intervention: Using a system based on ML algorithms to assess blood loss.
Comparison: Assessing blood loss using traditional methods.
Outcome: Including but not limited to the correlation between different measurement methods.

### 2.3 Eligibility Criteria

Studies were included if they met the following criteria: Studies evaluating the effectiveness of using ML models in intraoperative bleeding assessment; subjects were surgical patients (including orthopedic, general surgery, obstetrics and gynecology, and urology); study aim was to evaluate blood loss using ML‐based algorithms; outcome measures were correlation coefficients between blood loss estimates obtained by ML models and those obtained by gold‐standard methods.

Studies were excluded if they were dissertations, reviews, meta‐analyses, commentaries, newspaper articles, letters, duplicate publications, incomplete or inaccessible, or published in languages other than English. Conference papers are excluded due to low data reliability.

### 2.4 Search Strategy and Selection Criteria

We systematically searched for relevant literature using Web of Science, PubMed, Embase, the Cochrane Library, and CINAHL. The searches were conducted from the establishment of each database up to January 31, 2025, and later updated to include the period from February 1, 2025, to August 18, 2025. Additionally, we manually searched the reference lists of the identified articles and previous reviews to identify additional publications. We also searched clinical trial registries (such as ClinicalTrials.gov, clinicaltrialregister.eu) and preprint platforms (such as medRxiv, arxiv). The core search strategy involved two groups of terms combined with Boolean operators (Supplementary Table S1).

Two researchers specializing in evidence‐based nursing independently screened the literature, extracted data, and evaluated the included studies’ quality. Disagreements were resolved through discussion, with a third researcher making the final decision if needed. First, duplicates were excluded. The titles and abstracts were read to exclude irrelevant studies. Finally, the full texts were reviewed, and studies were selected and included based on the inclusion and exclusion criteria. Included studies were independently extracted using a predesigned Excel extraction sheet. The extracted information included the first author, publication year, country, study design, study subjects, sample type (simulated samples or real clinical samples), sample size, modeling method, gold standard, correlation coefficient, and whether an external validation was conducted.

### 2.5 Quality Assessment

The quality of the included studies was evaluated using the Newcastle‐Ottawa Scale (NOS) (Stang 2010). This scale includes three dimensions–selection of the study population, comparability, and exposure/outcome–with a total of eight items. A semi‐quantitative “star system” was used for evaluation, with a maximum score of 9 points. Except for “comparability,” which can receive up to 2 stars, all other items are rated with a maximum of 1 star. A higher score indicates higher study quality, and a score of ≥8 points indicates a low risk of bias.

### 2.6 Statistical Analysis

Meta‐analysis of the correlation coefficients was performed using Stata 17.0 software. We extracted the Pearson correlation coefficient as the primary effect indicator. Two of the literature reports provided the coefficient of determination *R* ^2^ (Li et al. 2023, 2020), which was extracted after conversion using the formula (Field 2018). Pearson correlation coefficients (*r*) were extracted from the included studies and converted into Fisher's *Z* values using the formula Fisher's $Z=\frac{1}{2}\ln\frac{1+r}{2-r}$. The 95% confidence interval of the transformed Fisher's Z value was calculated. Fisher's *Z* values were used as the effect size for the meta‐analysis, and the inverse Fisher's *Z* formula $r=\frac{e^{2Z}-1}{e^{2Z}+1}$ was used to revert to the correlation coefficient (*r*) and its 95% confidence interval (Summary *r*). Heterogeneity was assessed using the *I* ^2^ test. If *I* ^2^ < 50%, low heterogeneity was indicated, and a fixed‐effect model was used for combining; if *I* ^2^ > 50%, high heterogeneity was indicated, and a random‐effects model was used. If significant heterogeneity was observed (*I ^2^* > 50%), meta regression was further used to analyze the source of heterogeneity, and subgroup analyses were performed based on the meta‐regression results. It is important to note that several included studies (e.g., Li et al. 2020, 2023; Konig et al. 2014, 2018a) reported more than one correlation coefficient derived from testing different ML algorithms or under varying experimental conditions (e.g., lighting) within the same patient or sample cohort. These effect sizes are therefore not statistically independent. To incorporate all available data while acknowledging this dependency, all reported effect sizes from such studies were treated as separate entries in the primary meta‐analysis. This approach maximizes data utilization but may slightly underestimate the standard errors of the pooled estimates because within‐study correlations are not accounted for. To assess the robustness of our findings, we conducted a sensitivity analysis in which only one effect size (the one from the best‐performing algorithm or the most representative condition) from each of these studies was selected, and the meta‐analysis was repeated. The results of this sensitivity analysis are reported alongside the primary analysis. Deek's funnel plot and Egger's test were used to assess publication bias.

We categorized the included literature into three groups by year: 2014–2017, 2018–2020, and 2021–2023. During 2014–2017, ML algorithms were initially applied to medical image analysis, and during 2018–2020, with the standardization of electronic health records, the quality of medical AI datasets improved significantly. From 2021 to 2023, with the rise of the Transformer architecture and the maturation of multimodal fusion technology, the demand for model evaluation shifted to computational real‐time performance (Esteva et al. 2017; Mandel et al. 2016). We distinguished between China and the United States because there are significant differences between them in terms of healthcare systems, hospital hierarchical structures, clinical practice guidelines, and patient population characteristics, potentially affecting the primary outcome measures (Yip et al. 2019). Patients were divided into “cesarean section” and “other surgical patients” groups due to the presence of amniotic fluid potentially interfering with the assessment of bleeding outcomes. Samples were divided into “simulated” and “clinical”; simulated samples are highly controllable but lack physiological compensatory interference; meanwhile, real samples contain confounding factors (coagulation function, drug interference), which are critical for clinical translation validation (Sokol et al. 2025). The classification into internal and external validation was based on the validation method; internal validation (data split within the same institution) carries the risk of overfitting, whereas external validation is the core standard for clinical applicability (Wynants et al. 2019). Modeling methods were categorized into the Triton method, classical ML, and deep learning, as these three methods have fundamental differences in computational architecture. The Triton method relies on physical models and requires manually designed light absorption formulas; classical ML relies on manual feature selection, whereas deep learning enables end‐to‐end feature learning, with differences in computational efficiency (Shen et al. 2021).

It is worth noting that the logistic regression model was classified as a classical ML predictive model in this study rather than a traditional clinical assessment method (which does not rely on constructing predictive algorithm models but primarily depends on healthcare professionals’ judgments or direct physical measurements of blood loss, including visual estimation, weighing, volumetric, and Hb methods). In this study, any computational method that used algorithms to learn patterns from input data (such as patient demographic characteristics, preoperative laboratory indicators, intraoperative vital signs, surgical parameters, and imaging features) and output predictions of intraoperative blood loss was classified as an ML method. Logistic regression models used input features to learn weight parameters through mathematical optimization, generating a predictive function to output the probability of intraoperative blood loss reaching a certain level, which meets our defined ML method criteria (Hahn et al. 2016).

## 3 Results

### 3.1 Study Selection

**FIGURE 1:**
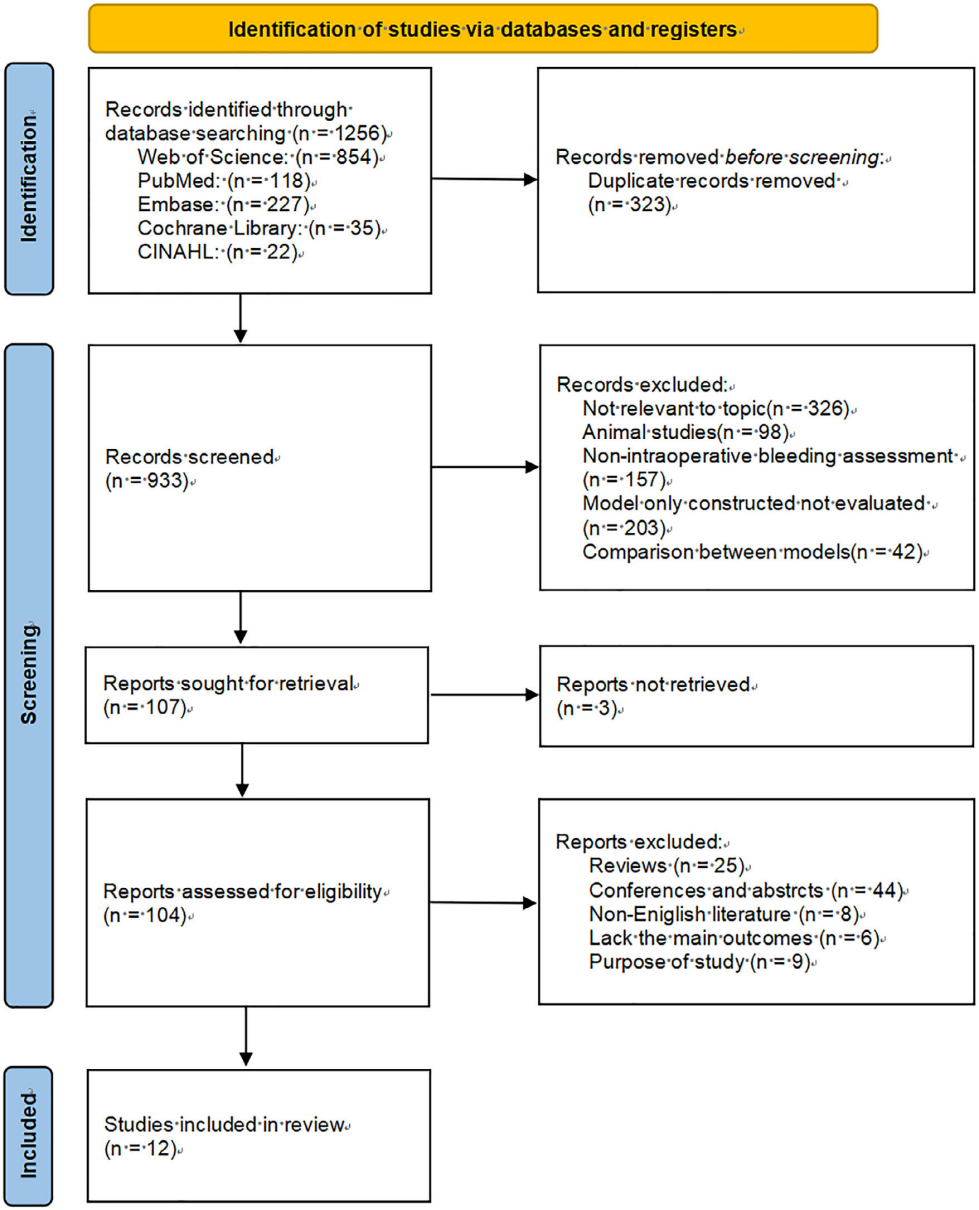
PRISMA flowchart of study selection.

The initial search identified 1256 articles. After removing duplicates, 933 articles remained. A total of 826 articles were excluded after title (326 not relevant to the topic, 98 animal studies, 157 not‐intraoperative bleeding assessment, 203 model only constructed not evaluated, 42 comparisons between models) and abstract reviews and 95 after full‐text reviews (3 not retrieved, 25 reviews, 44 conferences and abstracts, 6 non‐English literature, 6 lack of main outcomes, 9 incompatible purpose of study), and the remaining 12 (Holmes et al. 2014; Konig et al. 2014, 2018a, 2018b; Sharareh et al. 2015; Doctorvaladan et al. 2017; Nowicki et al. 2018; Fedoruk et al. 2019; Rubenstein et al. 2019; Saoud et al. 2019; Li et al. 2020, 2023) met the eligibility criteria (Figure 1).

### 3.2 Basic Characteristics

The studies were primarily published between 2014–2023. Two studies (Li et al. 2020, 2023) were conducted in China. One study (Rubenstein et al. 2019) was retrospective. Four studies (Doctorvaladan et al. 2017; Fedoruk et al. 2019; Rubenstein et al. 2019; Saoud et al. 2019) involved cesarean section patients, two studies (Sharareh et al. 2015; Nowicki et al. 2018) involved orthopedic surgery patients, and six studies (Holmes et al. 2014; Konig et al. 2014, 2018a, 2018b; Li et al. 2023, 2020) did not specify the type of surgery. Four studies (Konig et al. 2014, 2018a; Li et al. 2023, 2020) reported using expired donated whole blood, concentrated red blood cells, plasma, and saline to simulate blood loss, whereas eight studies (Holmes et al. 2014; Sharareh et al. 2015; Doctorvaladan et al. 2017; Konig et al. 2018b; Nowicki et al. 2018; Fedoruk et al. 2019; Rubenstein et al. 2019; Saoud et al. 2019) used real clinical samples. Ten studies (Holmes et al. 2014; Konig et al. 2014, 2018a, 2018b; Sharareh et al. 2015; Doctorvaladan et al. 2017; Nowicki et al. 2018; Fedoruk et al. 2019; Rubenstein et al. 2019; Saoud et al. 2019) used the Triton system, and the remaining two studies (Li et al. 2023, 2020) used multiple image feature extraction‐based modeling methods, including linear regression, random forest, XGBoost, DenseNet, YOLOv5, ResNet‐50, and SE‐ResNet50. Two studies (Sharareh et al. 2015; Nowicki et al. 2018) used spectrophotometry as the gold standard, whereas 10 studies (Holmes et al. 2014; Konig et al. 2014, 2018a, 2018b; Doctorvaladan et al. 2017; Fedoruk et al. 2019; Rubenstein et al. 2019; Saoud et al. 2019; Li et al. 2023, 2020) used the Hb method. In terms of validation methods, two studies (Li et al. 2023, 2020) used only internal validation, whereas the other 10 studies (Holmes et al. 2014; Konig et al. 2014, 2018a, 2018b; Sharareh et al. 2015; Doctorvaladan et al. 2017; Nowicki et al. 2018; Fedoruk et al. 2019; Rubenstein et al. 2019; Saoud et al. 2019) used external validation methods. The basic characteristics of the included studies are presented in Table 1.

**TABLE 1:** Summary of Included Studies' Characteristics.

Included Study	Country	Study Design	Study Subjects	Sample Type	Sample Size	Modeling Method	Gold Standard	Correlation Coefficient *r*	External Validation
Li et al. (2020)	China	Prospective	Surgical patients	Blood‐simulated surgical sponges (simulated samples)	569	LR RF XGBoost DenseNet	Hb method	LR 0.95 RF 0.97 XGBoost 0.97 DenseNet 0.98	Internal
Doctorvaladan et al. (2017)	USA	Prospective	Cesarean section women	Surgical sponges, suction bottles (real clinical samples)	757	Triton	Hb method	0.95	External
Rubenstein et al. (2019)	USA	Retrospective	Cesarean section women	Surgical sponges, suction bottles (real clinical samples)	2781	Triton	Hb method	0.72	External
Fedoruk et al. (2019)	USA	Prospective	Cesarean section women	Surgical sponges, suction bottles (real clinical samples)	61	Triton	Hb Method	0.33	External
Holmes et al. (2014)	USA	Prospective	Surgical patients	Surgical sponges, suction bottles (real clinical samples)	758	Triton	Hb method	0.93	External
Konig et al. (2018a)	USA	Prospective	Surgical patients	Saline + blood‐simulated suction bottles (simulated samples)	621	Triton	Hb method	Bright 0.41, Medium 0.36, Dark 0.35	External
Konig et al. (2014)	USA	Prospective	Surgical patients	Saline + blood‐simulated suction bottles (simulated samples)	621	Triton	Hb method	Bright 0.41, Medium 0.36, Dark 0.35	External
Nowicki et al. (2018)	USA	Prospective	Orthopedic surgery patients	Surgical sponges, suction bottles (real clinical samples)	781	Triton	Spectrophotometry	0.94	External
Sharareh et al. (2015)	USA	Prospective	Orthopedic surgery patients	Surgical sponges (real clinical samples)	857	Triton	Spectrophotometry	0.92	External
Li et al. (2023)	China	Prospective	Surgical patients	Surgical sponges, suction bottles (simulated samples)	851	YOLOv5 ResNet‐50 SE‐ResNet50	Hb method	YOLOv5 0.98 ResNet‐50 0.99 SE‐ResNet50 0.99	Internal
Konig et al. (2018b)	USA	Prospective	Surgical patients	Surgical sponges (real clinical samples)	797	Triton	Hb method	0.93	External
Saoud et al. (2019)	USA	Prospective	Cesarean section women	Surgical sponges, suction bottles (real clinical samples)	242	Triton	Hb method	0.26	External

*Notes*: (1) LR, Linear regression; RF, Random forest; XGBoost, eXtreme Gradient Boosting; YOLOv5, You Only Look Once version 5; SE‐ResNet50, Squeeze‐and‐Excitation Residual Network 50; Triton, Triton Inference Server; Hb, hemoglobin.

(2) Sample size is the sum of surgical sponges and suction bottles.

(3) External validation refers to evaluating a model using a dataset that is completely independent from the training dataset and was not used in the model's development process. Since this dataset does not overlap with the training dataset, it better reflects the model's generalization ability.

(4) The Hb method refers to measuring Hb loss by manually rinsing the red blood cell content from the sponges (mHbRinse). Sponges were soaked in heparinized normal saline and rinsed individually using a manual compression device. After rinsing, the Hb concentration of the effluent solution (HbEffluent) was measured using a low‐concentration Hb analyzer. The mass of the effluent solution was measured using a digital scale and converted to volume (VEffluent) assuming a mean fluid density of 1.0 g/mL. The Hb mass in the effluent was then calculated as: mHbEffluent = HbEffluent × VEffluent. The accuracy of this method has been verified using sponges stained with known amounts of blood (Holmes et al. 2014).

### 3.3 Quality Assessment

After evaluation by the two reviewers, all 12 studies included in this review were rated as having low risk of bias (Supplementary Table S2). Five studies (Holmes et al. 2014; Konig et al. 2014; Sharareh et al. 2015; Doctorvaladan et al. 2017; Li et al. 2020) scored only one point in the comparability aspect because they did not adjust for key confounding factors of the included patients (Supplementary Table S2).

### 3.4 Meta‐Analysis

**FIGURE 2:**
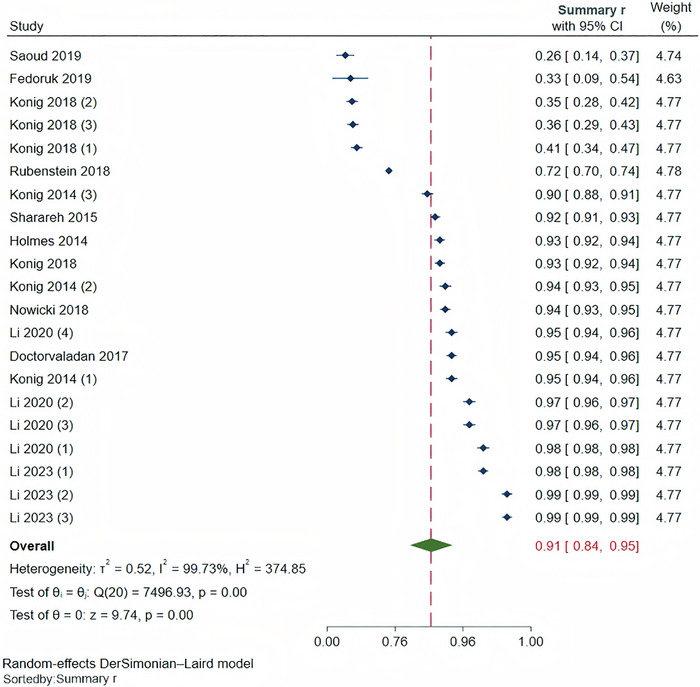
Forest plot of ML algorithm for assessing intraoperative blood loss. Note. Li et al. (2020) 1, 2, 3, and 4 represent LR, RF, XGBoost, and DenseNet data, respectively. Konig et al. (2018a) and Konig et al. (2014) 1, 2, and 3 represent data under bright, medium, and dark conditions, respectively. Li et al. (2023) 1, 2, and 3 represent YOLOv5, ResNet‐50, and SE‐ResNet50 data, respectively.

Heterogeneity was high (*I* ^2^ = 99.73%, *p* < 0.001), so a random‐effects model was used (Figure 2). The results showed a strong correlation between the ML‐based model evaluation of blood loss and the gold standard (*r* = 0.91; 95% confidence intervals [CI], 0.84 to 0.95). A sensitivity analysis was conducted by sequentially removing individual studies (Supplementary Figure S1). The results showed that the correlation coefficient for the ML algorithm in evaluating the intraoperative blood loss ranged from 0.90 to 0.92, which was similar to the overall correlation coefficient, indicating the stability of the study results. Furthermore, to assess the robustness of analyses that incorporated multiple effect sizes from individual studies, we reanalyzed the data by selecting only the best‐performing algorithm from each such study. The resulting pooled correlation coefficient was 0.90 (95% CI, 0.82 to 0.94), which was consistent with the findings of the primary analysis.

### 3.5 Publication Bias

Visual inspection of the funnel plot suggested possible publication bias, confirmed by Egger's test (*p* = 0.042) (Supplementary Figure 2). The “trim and fill” method was used to adjust the analysis for the effects of publication bias (Duval and Tweedie 2000). The uncorrected *r* was 0.91 (95% CI, 0.84 to 0.95), whereas the corrected *r* was 0.89 (95% CI, 0.79 to 0.94).

### 3.6 Meta‐Regression Results

A univariate meta‐regression analysis was performed using publication year, country, study subject, sample type, modeling method, and external validation as covariates (Supplementary Table S3). The results showed that the regression results for publication year (β = 0.174, *p* = 0.003), country (β = 2.710, *p* = 0.028), study subject (β = −0.061, *p* = 0.009), and modeling method (β = −0.216, *p* < 0.001) were statistically significant.

### 3.7 Subgroup Analysis

The results of the subgroup analysis are shown in Supplementary Table S4. Studies published between 2014 and 2017 showed that Summary *r* was 0.86 (95% CI, 0.75 to 0.92; *I^2^* = 99.62%; *p* < 0.001) for the ML algorithm and the gold standard, which was lower than the Summary *r* for studies published between 2018 and 2020 (*r* = 0.93; 95% CI, 0.91 to 0.95; *I^2^* = 88.94%; *p* = 0.003) and those published between 2021 and 2023 (*r*, 0.99; 95% CI, 0.98 to 0.99; *I^2^* = 99.48%; *p* < 0.001). Studies from China (*r* = 0.98; 95% CI, 0.97 to 0.99; *I^2^* = 98.40%; *p* < 0.001) showed a higher correlation than studies from the United States (*r* = 0.82; 95% CI, 0.70 to 0.90; *I^2^* = 99.58%; *p* < 0.001). The correlation between the ML algorithm and gold standard was lower in cesarean section patients (*r* = 0.69; 95% CI, 0.20 to 0.90; *I^2^* = 99.57%; *p* < 0.001) than in other surgical patients (*r* = 0.93; 95% CI, 0.86 to 0.97; *I^2^* = 99.74%; *p* < 0.001). Studies using simulated samples showed a higher correlation (*r* = 0.94; 95% CI, 0.85 to 0.97; *I^2^* = 99.78%; *p* < 0.001) compared with studies using real clinical samples (*r* = 0.85; 95% CI, 0.72 to 0.92; *I^2^* = 99.49%; *p* < 0.001). Studies using the Triton modeling system showed the lowest correlation (*r* = 0.82; 95% CI, 0.70 to 0.90; *I^2^* = 99.58%; *p* < 0.001), followed by studies using classical ML methods (*r* = 0.97; 95% CI, 0.96 to 0.98; *I^2^* = 95.15%; *p* < 0.001), and studies using deep learning models (*r* = 0.99; 95% CI, 0.98 to 0.99; *I^2^* = 97.10%; *p* < 0.001). Studies using internal validation showed a higher correlation (*r* = 0.98; 95% CI, 0.97 to 0.99; *I^2^* = 98.40%; *p* < 0.001) compared with studies using external validation (*r* = 0.82; 95% CI, 0.70 to 0.90; *I^2^* = 99.59%; *p* < 0.001).

## 4 Discussion

### 4.1 Overall Low Risk of Bias in Included Studies and Good Model Performance

The 12 included studies were well designed, with NOS scores ranging from 8 to 9, indicating low overall risk of bias. The pooled correlation coefficient was 0.91 (95% CI, 0.84 to 0.95; *I^2^* = 99.73%; *p* < 0.001), suggesting that ML‐based models can effectively assess intraoperative blood loss. Previous studies confirm ML systems assess blood loss more accurately than conventional methods. Doctorvaladan et al. (2017) found that the correlation between the results from the Triton system and actual blood loss was higher than that of visual estimation and the widely used quantitative weighing method. Holmes et al. (2014) similarly found that the Triton system provided higher accuracy and greater consistency than the weighing method when measuring blood loss during abdominal surgery. Models based on different algorithms demonstrate varying performances across different datasets. Li et al. (2020) compared four algorithmic models for accuracy in assessing surgical blood loss and found that methods based on DenseNet and XGBoost achieved the best performance in terms of blood loss and Hb loss, with the DenseNet model showing a higher 95% CI.

Despite high correlation, heterogeneity was significant (*I* ^2^ = 99.73%, *p* < 0.001). A meta‐regression analysis was conducted to quantify the contributions of various factors to heterogeneity, revealing that “publication year,” “country,” “study population,” and “modeling method” were significant sources of heterogeneity (*p* < 0.05), whereas “sample type” and “external validation” were not (*p* > 0.05). Country made the most significant contribution (β = 2.71, *p* = 0.028), with studies from the United States showing significantly higher effect sizes than those from other countries. Study population was another important factor (β = −0.061, *p* = 0.009), with studies targeting cesarean section patients generally yielding lower accuracy estimates than those targeting other surgical patients. However, subgroup analysis revealed that heterogeneity remained extremely high (*I^2^* > 95%) across all defined subgroups, indicating that it is driven by complex, multifactorial interactions, potentially including unmeasured study characteristics or inherent, unexplained random variability.

Sensitivity analysis demonstrated that the study results were stable. However, the funnel plot and Egger's test indicated potential publication bias. After further *T*–*F* test correction, the result was *r* = 0.89 (95% CI, 0.79 to 0.94), which was similar to the uncorrected result. This suggests that, despite some publication bias, the overall results of the study remain robust. Overall, the ML algorithms demonstrated high accuracy and reliability in assessing intraoperative blood loss.

Although this meta‐analysis showed a high pooled correlation between ML estimates and reference standards (*r* = 0.91), interpretation of this finding requires caution. First, the extremely high heterogeneity (*I^2^* = 99.73%) indicates substantial variability among the included studies. Therefore, this pooled correlation coefficient should be interpreted as a general indicator of the strength of association within the existing evidence rather than as a uniform performance metric directly applicable across different clinical settings. Second, it is essential to distinguish between “correlation” and “agreement.” High correlation reflects a consistent trend between ML estimates and actual blood loss, but does not directly quantify absolute measurement accuracy. For example, even with a high correlation, a model may systematically overestimate or underestimate blood loss within specific bleeding ranges. Ideally, evaluation should be supplemented with agreement analyses (e.g., Bland–Altman plots) to quantify the magnitude and limits of estimation error. However, such data were generally unavailable in the studies included in this review. Finally, the subgroup analyses and overall limitations of this study highlight substantial gaps in the evaluation of massive bleeding (>1500 mL), which represents the most critical scenario for clinical decision‐making. Future studies should prioritize assessing model performance in high‐volume bleeding settings and reporting agreement metrics to ensure reliability and safety across varying degrees of hemorrhage, particularly in life‐threatening cases, thereby strengthening the evidence base for clinical implementation.

### 4.2 Multiple Factors Affect the Performance of ML Models in Predicting Intraoperative Blood Loss

Subgroup analysis further explored the influence of various factors on the performance of the ML algorithms in assessing intraoperative blood loss. The results showed that factors such as publication year, country, study subjects, and modeling methods significantly affected the effectiveness of the ML models. Studies published between 2021 and 2023 had the highest accuracy, likely due to technological advancements and improved data quality. Subgroup analyses revealed differences in model performance across countries. We suspect that these variations stem from factors such as data collection and preprocessing procedures, composition of surgical cases, diversity of training data, and approaches used for model validation. For instance, in some Chinese studies (Li et al. 2023, 2020), deeper image feature extraction algorithms were employed, showing higher correlations under internal validation, whereas several other studies (Holmes et al. 2014; Konig et al. 2014, Konig et al. 2018a, 2018b; Sharareh et al. 2015; Doctorvaladan et al. 2017; Nowicki et al. 2018; Fedoruk et al. 2019; Rubenstein et al. 2019; Saoud et al. 2019) relied on the Triton system or external validation, potentially introducing additional heterogeneity. The accuracy of the Triton system for assessing intraoperative blood loss is affected by illumination conditions (Konig et al. 2014), whereas external datasets often differ in terms of sample preprocessing, imaging conditions, or patient demographics (DeGrave et al. 2021). Ensuring consistency in data labeling and acquisition protocols is crucial for minimizing these variations and improving the real‐world model performance. It is recommended that future multicenter studies be conducted to establish standardized protocols, with researchers at each center adopting uniform operating procedures to ensure the stability of external result validation (Kelly et al. 2019).

The accuracy of ML algorithms for assessing blood loss varies across different types of surgeries. This study found that the correlation between ML‐based blood loss assessment and the gold standard was the weakest in cesarean section patients. Postpartum hemorrhage is a major cause of maternal mortality during pregnancy, accounting for 27% of maternal deaths (Doctorvaladan et al. 2017; Liu et al. 2021). Owing to factors such as uterine incisions, placental issues, trauma to the birth canal, or the mother's own coagulation dysfunction, the risk of postpartum hemorrhage is higher in women who undergo cesarean section than in those who undergo vaginal delivery (Nieto‐Calvache et al. 2025). Therefore, accurate assessment of blood loss in cesarean sections is particularly important. A key limitation of current ML models is their inability to distinguish between blood and amniotic fluid when estimating blood loss (Li et al. 2023), leading to measurement errors. Hemorrhage management guidelines (Obstetrics Subgroup, Chinese Society of Obstetrics and Gynecology, Chinese Medical Association Chinese Society of Perinatal Medicine, Chinese Medical Association 2023) highlight that the presence of amniotic fluid is a key factor affecting the accuracy of blood loss assessment during cesarean sections. Therefore, although ML‐based predictions of blood loss in patients undergoing cesarean section show good predictive performance, their clinical applicability requires further evaluation.

Models constructed using deep learning methods outperformed the classical ML models and the Triton system. The Triton system is a colorimetric‐based mobile application certified by the U.S. Food and Drug Administration (Park et al. 2025). It captures images of surgical sponges during surgery using a tablet's built‐in camera and then utilizes cloud‐based ML technology to provide real‐time and accurate estimations of Hb content on the sponges. It is worth noting that the detailed algorithm used by the Triton system to estimate Hb loss is still in the optimization phase, and its accuracy has room for improvement. Konig et al. (2014) showed that the accuracy of the Triton system in assessing intraoperative blood loss is influenced by lighting conditions, with *R* ^2^ values decreasing in dark, medium, and bright lighting environments by 0.165, 0.131, and 0.125, respectively. Additionally, the system indirectly measures blood loss by calculating Hb loss and using a formula to estimate the blood volume. In contrast, advanced deep learning approaches (e.g., DenseNet and ResNet50) can directly identify hemorrhagic regions and classify fluid types, such as blood versus irrigation fluid, thereby achieving higher accuracy than purely colorimetric‐based systems. Our analysis reveals a critical trade‐off among the three approaches. Classical ML models offer superior interpretability, crucial for nursing trust, but may lack robustness. Deep learning models promise higher accuracy but function as “black boxes,” posing a significant barrier to clinical adoption without explainable AI (XAI) techniques. The Triton system, as an integrated solution, prioritizes workflow integration and regulatory approval over algorithmic transparency. For optimal deployment in nursing, the ideal system must balance this triad of accuracy, explainability, and seamless workflow integration to become a trustworthy aid rather than a disruptive nuisance (Amann et al. 2020; Ronquillo et al. 2021b).

This study investigated the sources of heterogeneity through subgroup analysis and meta‐regression. Notably, although subgroup analysis suggested that “sample type” and “external validation” might be potential influencing factors, the results of meta‐regression indicated that these factors did not contribute significantly to heterogeneity after adjusting for other covariates (e.g., country, modeling method) (*p* > 0.05). This seemingly inconsistent result may stem from multicollinearity among variables (Schmid and Stijnen 2021). For example, in the literature included in this study, the majority of “real clinical sample” studies were conducted in the United States and utilized the Triton modeling method, whereas “simulated sample” studies encompassed a broader range of countries and methodologies. Therefore, the between‐group differences observed in the subgroup analysis are likely driven primarily by the strong confounding factors of “country” and “modeling method,” rather than the independent effects of “sample type” or “external validation” themselves. The meta‐regression model effectively isolates and assesses the independent contribution of each factor by simultaneously incorporating all variables, yielding more reliable results. In summary, we believe that “publication year,” “country,” “study population,” and “modeling method” are the primary independent sources of heterogeneity in this meta‐analysis. The associations observed for “sample type” and “external validation” are more likely due to confounding effects with other variables.

Although we identified statistically significant sources of heterogeneity through meta‐regression analysis, including publication year, country, study population, and modeling method (*p* < 0.05), it should be explicitly noted that *I^2^* remained consistently above 95% across all subgroup analyses. This finding indicates that the identified factors explain only a portion of the observed heterogeneity, with substantial residual variability remaining unaccounted for. Accordingly, the pooled correlation coefficient (*r* = 0.91) reported in this meta‐analysis should be interpreted cautiously as a general description of the strength of association within the existing evidence, rather than as a stable and uniform performance metric applicable across diverse clinical settings. Potential unmeasured contributors to this persistently high heterogeneity may include, but are not limited to (1) subtle differences in data collection and preprocessing, such as variation in imaging equipment, operating room lighting standardization, and workflows for processing intraoperative gauze or suction bottle images; (2) heterogeneity in algorithm implementation and integration, including differences in model architecture, hyperparameter selection, training strategies, and the extent of integration into clinical workflows; (3) micro‐level clinical context differences, whereby patient‐specific factors (e.g., baseline anemia and coagulation status), intraoperative medications (e.g., vasoactive agents), and temporal variations in bleeding rate and pattern may influence model performance; and (4) variability in reference standard execution, as even when Hb measurement is used as the reference standard, differences in irrigation techniques, device calibration, and measurement precision may introduce additional variability. This residual, extremely high heterogeneity highlights a fundamental challenge in translating ML models for intraoperative blood loss assessment: model performance is highly dependent on the specific environment in which the model is developed and validated. These findings highlight the need for future research to move beyond reporting average performance metrics and to prioritize standardized data collection protocols, development of more robust and generalizable algorithms, and rigorous external validation to assess performance across diverse clinical settings.

Our meta‐analysis indicates that ML models can achieve high accuracy in estimating blood loss. Beyond technical performance, the potential to translate this technology into clinical practice holds promise across multiple key areas, with the potential to revolutionize perioperative care. The accuracy of real‐time quantification of blood loss provided by ML models surpasses that of subjective visual estimates. This provides nurses with objective data to support critical decisions. For example, when the system automatically issues an alert for sustained, above‐average bleeding, nurses can take the following actions: (1) prioritize more frequent vital sign monitoring; (2) initiate protocol‐based interventions, such as fluid resuscitation or preparation of blood products; (3) report abnormal conditions to surgeons and anesthesiology teams earlier than when relying solely on traditional hemodynamic instability signs (Ronquillo et al. 2021a). Integrating automated estimation tools into electronic health records reduces cognitive burden and manual documentation time (e.g., weighing gauze, calculating volumes). This allows perioperative nurses to reallocate valuable time to direct patient care and monitoring, ultimately improving efficiency and reducing the risk of documentation errors. The core value of this technology lies in its potential for early warning. By objectively identifying occult bleeding or confirming significant blood loss, ML models can serve as the foundation of a proactive safety system. This enables nursing and surgical teams to intervene before patients progress to overt hemodynamic compromise, potentially reducing the incidence of hypovolemic shock and related complications (Booth et al. 2021).

### 4.3 Implications for Future Research

This study had some limitations. First, although the quality of the included studies was high, individual studies (Fedoruk et al. 2019; Saoud et al. 2019) had small sample sizes, and most studies included patients with blood loss within the normal range, which may have affected the assessment of model performance. Patients with blood loss exceeding 1500 mL were underrepresented, which somewhat limited the generalizability of the results. Second, publication bias may have affected the accuracy of the study findings. Additionally, owing to the high heterogeneity of the included studies, the results of the subgroup analysis should be interpreted with caution. Finally, this study included only Chinese and English‐language literature, potentially introducing language selection bias. Consequently, important studies from other regions may have been omitted, affecting the comprehensiveness and representativeness of the systematic review. Future research should aim to include literature published in other languages to enhance the global applicability of the findings.

Future research should refine population stratification and develop models specific to surgery type, disease, and demographics. Additionally, testing time should be included in the outcome to assess the ML speed advantage. Moreover, attention should be paid to the clinical benefits of ML models, such as reducing the incidence of adverse outcomes and unnecessary blood transfusions. For instance, one study developed multiple ML classifiers to predict the need for intraoperative blood transfusions in patients undergoing non‐cardiac surgery. Among these, the logistic regression classifier performed the best, with an area under the receiver operating characteristic curve of 0.836, which was higher than that of the traditional multivariable regression models (Park et al. 2025). This suggests that ML models can more accurately predict transfusion requirements, thereby reducing unnecessary transfusions and lowering the risk of transfusion‐related complications. Additionally, some authors (Konig et al. 2018b) have proposed that the Triton system could be used to accurately measure blood loss during surgery, allowing for timely management of blood‐soaked sponges and early implementation of measures such as autologous blood transfusion, thus reducing the use of allogeneic blood products and conserving blood resources. Nevertheless, the accuracy of the model was limited by image quality, blood and non‐blood fluid mixing, and sample processing, suggesting that a standardized multimodal sample collection and processing procedure should be established for clinical use.

Strengthening the external validation component is an integral part of building model credibility, and it is crucial to accurately assess the generalization potential, robustness, and repeatability of the model, as well as to protect against overfitting (Audureau et al. 2025). Unfortunately, most of the ML prediction models included in this study lack this critical step, raising doubts about the generalizability of the reported ML models. In the future, when assessing the efficacy of ML models, it is important to emphasize extensive external validation across geographic regions and patient populations as a means of clarifying and consolidating the general applicability and stability of the models. This validation process should be accompanied by the continuous collection of feedback to guide the iterative optimization of the models to ensure that they perform as expected in different clinical scenarios.

The predictive models included in this study generally do not specify strategies for dealing with missing values, which, as an “unknown element” in the dataset, can weaken the overall integrity and reliability of the data if not handled appropriately. To address this issue, the following directions for improvement are worth exploring: first, we can consider introducing more accurate interpolation techniques, such as using generative adversarial networks for efficient interpolation, or exploring the potential of self‐coders in deep learning for missing value processing. In practice, missing data are often associated with non‐random factors, so future ML models should be developed to automatically identify and model these complex non‐random missing patterns, and accordingly select more relevant missing value processing means.

Although our ML models demonstrate high accuracy, their inherent complexity limits interpretability, posing obstacles to clinical trust and integration. Recognizing the “black box” nature of complex deep learning models such as DenseNet and SE‐ResNet, this study emphasizes that performance metrics alone are insufficient to support their clinical application, particularly in the field of nursing. The introduction of XAI technology is crucial for fostering trust and aiding clinical decision‐making (Gunning et al. 2019). In nursing practice, clinicians are responsible for overseeing algorithm outputs and integrating them into overall patient care, where understanding the “why” behind predictions is as important as the predictions themselves (Amann et al. 2020). Future research may focus on integrating post‐hoc explanation frameworks (such as Layered Relational Propagation or Grad‐CAM) to generate visualization highlight maps that intuitively display the most influential features in model predictions, enabling nursing colleagues to understand the algorithm.

Existing perioperative bleeding assessment methods suffer from low accuracy, cumbersome procedures that rely on staff expertise, and may lead to underestimation of bleeding volume, resulting in poor patient outcomes. Therefore, applying ML technology to clinical scenarios, and using computer vision technology to rapidly extract features from bleeding images—such as constructing a bleeding warning system through computer vision technology—can help assess patient blood loss volume, including microbleeding. Given these data, the system can assess risks such as postoperative active bleeding, rapidly issue warnings, and provide contingency plans for nurses to manage postoperative situations, helping improve outcomes and reduce workload. The results of this study may provide a reference for further exploring the application of artificial intelligence in other nursing fields, promoting the development of interdisciplinary research, and continuously advancing innovation and progress in the nursing discipline.

### 4.4 Implications for Nursing

Machine learning–based systems for intraoperative blood loss assessment have the potential to support nurses by providing more objective and timely estimates of bleeding than traditional visual or manual methods. Access to real‐time, automated blood loss information may enhance clinical judgement, support earlier recognition of abnormal bleeding, and facilitate timely communication with surgical and anaesthesia teams. By reducing reliance on subjective estimation and manual calculations, these tools may also help decrease cognitive workload and documentation burden for perioperative nurses, allowing greater focus on patient monitoring and safety. However, effective implementation will require appropriate training, clear clinical protocols, and careful consideration of how algorithm outputs are integrated into existing nursing workflows and decision‐making processes.

## 5 Conclusion

This study systematically summarizes the current evidence on ML algorithms for assessing intraoperative blood loss through a meta‐analysis. The results showed that ML models have high accuracy and reliability in the evaluation of intraoperative blood loss. Despite high heterogeneity and potential publication bias, the overall findings remained robust. Future studies should increase the sample size, standardize sample handling, refine the study population, and improve external validation. In addition, attention should be paid to testing time and true clinical benefit, and more accurate missing value handling techniques should be introduced.

## Author Contributions

All authors had full access to all data in the study and take responsibility for the integrity of the data and accuracy of the data analysis. Author contributions as follows: Conceptualization: Zhou, L. L. Pan, X. M. Pan, Rao. Methodology: Zhou, L. L. Pan. Validation: Zhou, X. M. Pan, Y. W. Li. Formal Analysis: Zhou, L. L. Pan, X. M. Pan, Y. W. Li. Investigation: Zhou, L. L. Pan, Y. W. Li. Data Curation: Rao. Writing – Original Draft: Zhou, L. L. Pan. Writing – Review and Editing: Zhou, L. L. Pan, X. M. Pan, Y. W. Li, H. Li. Visualization: Zhou, Y. W. Li. Supervision: Rao, H. Li. Project Administration: L. L. Pan, X. M. Pan. Funding Acquisition: Rao, H. Li

## Funding

This research was supported by the 2024 Shanghai Jiao Tong University Jiao Tong University Star Program Medical‐Industrial Intersection Research Fund (YG2024ZD26) and 2024 Health Management Excellent Young Talents Project of the Shanghai Rehabilitation Medical Association (2024JGYQ09).

## Ethics Statement

The study did not require a code of ethics as it did not include any human or animal subjects. The protocol was registered with PROSPERO (CRD42025642792).

## Supporting information




**Table S1**: PubMed Search Strategy.
**Table S2**: Quality Assessment of the Included Studies.
**Table S3**: Meta‐Regression Results.
**Table S4**: Subgroup Analysis of ML Models for Blood Loss Estimation.
**Figure S1**: Sensitivity Analysis Using the One‐Study Removal Method.
**Figure S2**: Deek's Funnel Plot for ML Algorithm in Assessing Intraoperative Blood Loss.

## Data Availability

The datasets analyzed during this study are available from the corresponding author on reasonable request.
