# Supplementary material for: Machine Learning in Assessing Intraoperative Blood Loss: A Systematic Review and Meta‐Analysis
Source: Int Nurs Rev. 2026 Feb 27;73(1):e70159. doi: 10.1111/inr.70159 (PMC12949346; doi:10.1111/inr.70159)
Supplement: Supplementary file 1 — Table S1: PubMed Search Strategy. Table S2: Quality Assessment of the Included Studies. Table S3: Meta‐Regression Results. Table S4: Subgroup Analysis of ML Models for Blood Loss Estimation. Figure S1: Sensitivity Analysis Using the One‐Study Removal Method. Figure S2: Deek's Funnel Plot for ML Algorithm in Assessing Intraoperative Blood Loss. [file INR-73-0-s001.docx]

**Tables**

**Supplementary Table S1. PubMed Search Strategy**

|  | Search Formulas |
| --- | --- |
| #1 | “Machine learning”[MeSH Terms] OR “Machine learning”[Title/Abstract] OR“Supervised Learning”[Title/Abstract] OR “Unsupervised Learning” [Title/Abstract] OR “Reinforcement Learning”[Title/Abstract] OR “Decision Tree”[Title/Abstract] OR “Neural Networks”[Title/Abstract] OR  “Support Vector Machine”[Title/Abstract] OR “Beyesian Model”[Title/Abstract] OR “Clustering”[Title/Abstract] OR “Reducing Dimensionality”[Title/Abstract] OR “Metric Learning”[Title/Abstract] OR “Ensemble Learning”[Title/Abstract] OR “Random Forest”[Title/Abstract] OR “Deep learning”[Title/Abstract] |
| #2 | “Postpartum Hemorrhage”[MeSH Terms] OR “Postpartum Hemorrhage”[Title/Abstract] OR “Obstetric Bleeding”[Title/Abstract] OR “Obstetric Hemorrhage”[Title/Abstract] OR “Primary Postpartum Hemorrhage”[Title/Abstract] OR “Immediate Postpartum Hemorrhage”[Title/Abstract] OR “Postnatal”[Title/Abstract] OR “Atonic Postpartum Hemorrhage”[Title/Abstract] OR “Hemorrhage”[MeSH Terms] OR “Surgical Hemoglobin Loss [Title/Abstract] OR “Surgical Blood Loss*”[Title/Abstract] OR “Surgical Hemorrhage*”[Title/Abstract] OR “Bleeding”[Title/Abstract] OR “Cardiac surgery”[Title/Abstract] OR “Valve replacement/repair”[Title/Abstract] OR “Vascular surgery”[Title/Abstract] OR “Angioplasty”[Title/Abstract] OR “Stent placement”[Title/Abstract] OR “Neurosurgery”[Title/Abstract] OR “Craniotomy”[Title/Abstract] OR “Spinal surgery”[Title/Abstract] OR “Orthopedic surgery”[Title/Abstract] OR “Joint replacement”[Title/Abstract] OR “Fracture repair”[Title/Abstract] OR “General surgery”[Title/Abstract] OR “Appendectomy”[Title/Abstract] OR “Cholecystectomy”[Title/Abstract] OR “Hernia repair”[Title/Abstract] OR “Obstetric/Gynecologic surgery”[Title/Abstract] OR “Cesarean section”[Title/Abstract] OR “Hysterectomy”[Title/Abstract] OR “Urologic surgery”[Title/Abstract] OR “Nephrectomy”[Title/Abstract] OR “Prostatectomy”[Title/Abstract] OR “Plastic surgery”[Title/Abstract] OR “Ophthalmic surgery”[Title/Abstract] OR “Transplant surgery”[Title/Abstract] OR “Oncologic surgery”[Title/Abstract] OR “Tumor resection”[Title/Abstract] OR “Pediatric surgery”[Title/Abstract] OR “Amputation”[Title/Abstract] |
| #3 | #1 AND #2 |

**Supplementary Table S2. Quality Assessment of the Included Studies**

| **Included Study** | **Selection of Study Population** | | | | **Comparability** | **Outcome** | | | **Total Score** | **Risk of Bias** |
| --- | --- | --- | --- | --- | --- | --- | --- | --- | --- | --- |
|  | **Exposure Group Representativeness** | **Non-Exposure Group Selection Method** | **Exposure Factor Determination** | **Outcome Indicator Determination** | **Group Comparability** | **Outcome Evaluation** | **Follow-up Duration** | **Completeness** |  |  |
| Li^[19]^ (2020) | * | * | * | * | * | * | * | * | 8 | low |
| Doctorvaladan^[20]^ (2017) | * | * | * | * | * | * | * | * | 8 | low |
| Rubenstein^[21]^ (2018) | * | * | * | * | ** | * | * | * | 9 | low |
| Fedoruk^[22]^ (2019) | * | * | * | * | ** | * | * | * | 9 | low |
| Holmes^[23]^ (2014) | * | * | * | * | * | * | * | * | 8 | low |
| Konig^[24]^ (2018a) | * | * | * | * | ** | * | * | * | 9 | low |
| Konig^[25]^ (2014) | * | * | * | * | * | * | * | * | 8 | low |
| Nowicki^[26]^ (2018) | * | * | * | * | ** | * | * | * | 9 | low |
| Sharareh^[27]^ (2015) | * | * | * | * | * | * | * | * | 8 | low |

**Supplementary Table S2. (Continued)**

| **Included Study** | **Selection of Study Population** | | | | **Comparability** | **Outcome** | | | **Total Score** | **Risk of Bias** |
| --- | --- | --- | --- | --- | --- | --- | --- | --- | --- | --- |
|  | **Exposure Group Representativeness** | **Non-Exposure Group Selection Method** | **Exposure Factor Determination** | **Outcome Indicator Determination** | **Group Comparability** | **Outcome Evaluation** | **Follow-up Duration** | **Completeness** |  |  |
| Li^[28]^ (2023) | * | * | * | * | ** | * | * | * | 9 | low |
| Konig^[29]^ (2018b) | * | * | * | * | ** | * | * | * | 9 | low |
| Saoud^[30]^ (2019) | * | * | * | * | ** | * | * | * | 9 | low |

Note: Newcastle-Ottawa Scale (NOS) uses a semi-quantitative “star system” for evaluation, 1 star represents a score.

Li 2020: Not adjusted for patient age or type of surgery; Doctorvaladan 2017: Not adjusted for patient age or gender; Holmes 2014: Not adjusted for patient age or type of surgery; Konig 2014: Not adjusted for preoperative hemoglobin levels and type of surgery; Sarareh 2015: Not adjusted for preoperative hemoglobin levels and type of surgery.

**Supplementary Table S3. Meta-Regression Results**

| **Subgroup** | **β** | **95% CI** | **P-value** | **SE** |
| --- | --- | --- | --- | --- |
| Publication Year | 0.174 | 0.058~0.290 | 0.003 | 0.059 |
| Country | 2.710 | 0.434~4.987 | 0.028 | 0.886 |
| Study Subjects | -0.061 | -0.107~-0.015 | 0.009 | 0.023 |
| Sample Type | 0.590 | -0.652~1.832 | 0.276 | 0.483 |
| Modeling Method | -0.216 | -0.307~-0.125 | <0.001 | 0.046 |
| External Validation | -0.326 | -2.447~1.795 | 0.709 | 0.825 |

**Supplementary Table S4. Subgroup Analysis of ML Models for Blood Loss Estimation**

| **Group** | **Number of Included Studies** | **Heterogeneity Test** | | **Effect Model** | **Meta-analysis Results** | | **r (95% CI)** |
| --- | --- | --- | --- | --- | --- | --- | --- |
|  |  | ***I^2^-value* (%)** | ***P-value*** |  | **Fisher’s Z (95% CI)** | ***P-value*** |  |
| Publication Year |  |  |  |  |  |  |  |
| 2014–2017 | 4^[20, 23, 25, 27]^ | 99.62 | <.001 | Random | 1.30 (0.98–1.62) | <.001 | 0.86 (0.75–0.92) |
| 2018–2020 | 7^[19, 21, 22, 24, 26, 29, 30]^ | 88.94 | .003 | Random | 1.66 (1.52–1.81) | <.001 | 0.93 (0.91–0.95) |
| 2021–2023 | 1^[28]^ | 99.48 | <.001 | Random | 1.84 (1.46–2.23) | <.001 | 0.99 (0.98–0.99) |
| Country |  |  |  |  |  |  |  |
| China | 2^[19, 28]^ | 98.40 | <.001 | Random | 1.16 (0.86–1.46) | <.001 | 0.98 (0.97–0.99) |
| US | 10^[20-27, 29, 30]^ | 99.58 | <.001 | Random | 2.27 (2.05–2.50) | <.001 | 0.82 (0.70–0.90) |
| Study Subjects |  |  |  |  |  |  |  |
| Cesarean Section Women | 4^[20-22, 30]^ | 99.57 | <.001 | Random | 0.84 (0.20–1.48) | .010 | 0.69 (0.20–0.90) |
| Other Surgical Patients | 8^[19, 23--29]^ | 99.74 | <.001 | Random | 1.70 (1.31–2.08) | <.001 | 0.93 (0.86–0.97) |
| Sample Type |  |  |  |  |  |  |  |
| Simulated Samples | 4^[19, 24, 25, 28]^ | 99.78 | <.001 | Random | 1.70 (1.25–2.15) | <.001 | 0.94 (0.85–0.97) |
| Real Clinical Samples | 8^[20-23, 26, 27, 29, 30]^ | 99.49 | <.001 | Random | 1.26 (0.91–1.61) | <.001 | 0.85 (0.72–0.92) |
| Modeling Method |  |  |  |  |  |  |  |
| Triton | 10^[20-27, 29, 30]^ | 99.58 | <.001 | Random | 1.16 (0.86–1.46) | <.001 | 0.82 (0.70–0.90) |
| Classical Machine Learning | 1^[19]^ | 95.15 | <.001 | Random | 2.26 (2.03–2.50) | <.001 | 0.97 (0.96–0.98) |
| Deep Learning | 1^[28]^ | 97.10 | <.001 | Random | 1.84 (1.46–2.23) | <.001 | 0.99 (0.98–0.99) |
| External Validation |  |  |  |  |  |  |  |
| Internal | 2^[19, 28]^ | 98.40 | <.001 | Random | 1.16 (0.86–1.46) | <.001 | 0.98 (0.97–0.99) |
| External | 10^[20-27, 29, 30]^ | 99.58 | <.001 | Random | 2.27 (2.05–2.50) | <.001 | 0.82 (0.70–0.90) |

**Figure Captions**


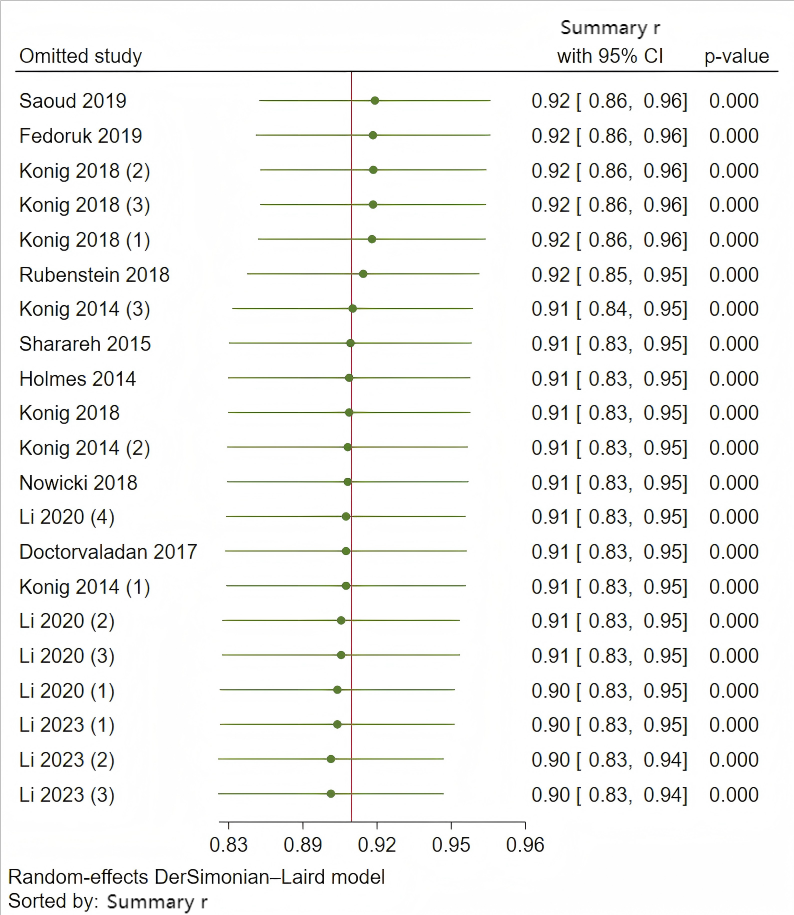


**Supplementary Fig1.** Sensitivity Analysis Using the One-Study Removal Method

**
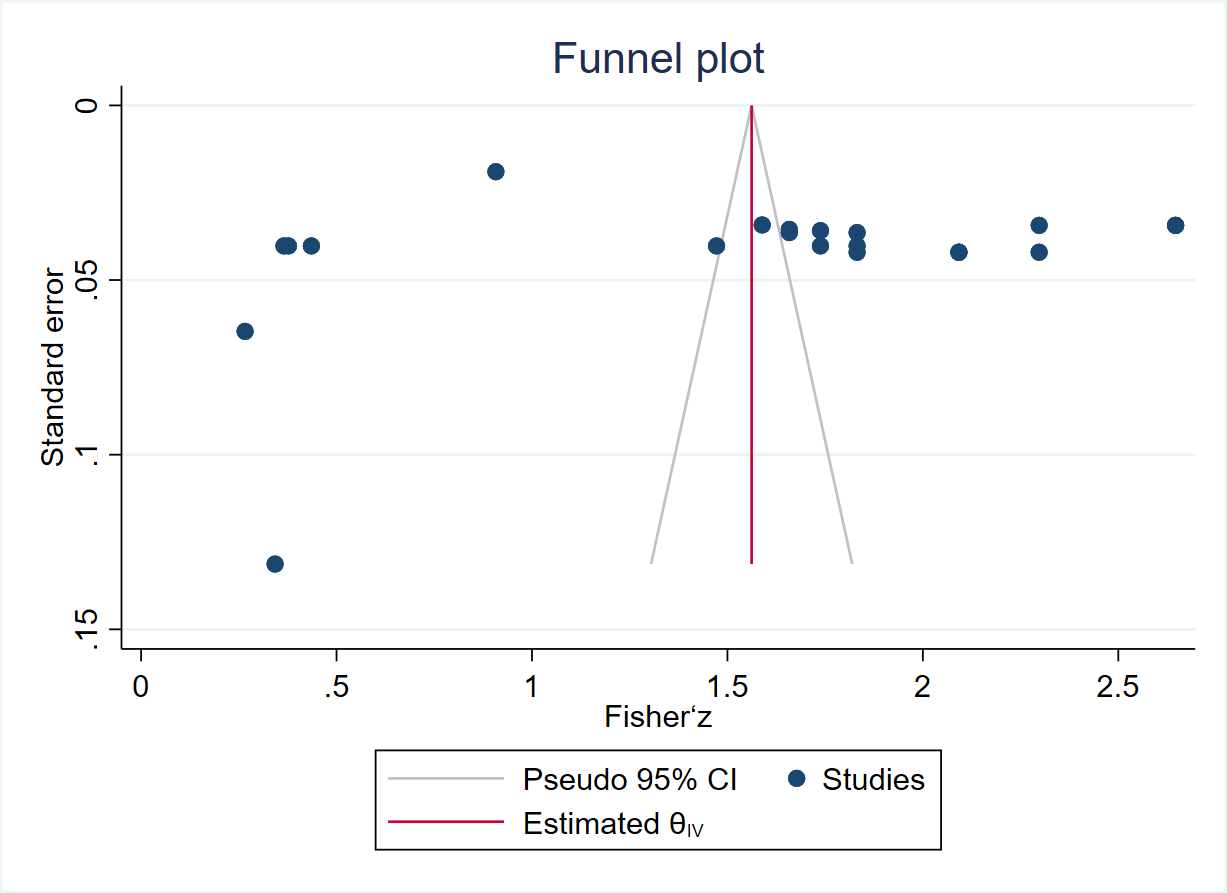
**

**Supplementary Fig2.** Deek's Funnel Plot for ML Algorithm in Assessing Intraoperative Blood Loss
